# The Associations between Exposure to Multiple Heavy Metals and Total Immunoglobulin E in U.S. Adults

**DOI:** 10.3390/toxics12020116

**Published:** 2024-01-30

**Authors:** Xin Song, Xiaowen Ding, Piye Niu, Tian Chen, Tenglong Yan

**Affiliations:** 1 Department of Occupational Health and Environmental Health, School of Public Health, Capital Medical University, Beijing 100069, China; songxin302@163.com (X.S.);; 2 Beijing Key Laboratory of Environmental Toxicology, Capital Medical University, Beijing 100069, China; 3 Beijing Institute of Occupational Disease Prevention and Treatment, Beijing 100093, China

**Keywords:** heavy metals, mixture, total immunoglobulin E (total IgE), BKMR

## Abstract

Immunoglobulin E (IgE) is a type of immunoglobulin, and elevated serum total IgE is often present in allergic diseases. Exposure to environmental heavy metals has been markedly linked to allergic diseases, leading to elevated total IgE levels. However, studies concerning the effects of multiple metal exposures on total IgE levels are limited. Therefore, the current study seeks to explore the correlation between heavy-metal co-exposure and total IgE levels based on the National Health and Nutrition Examination Survey (NHANES, 2005–2006). Participants possessed complete data on total IgE levels, 11 urinary metal concentrations and other covariates. The correlations between 11 metals and total IgE levels were analyzed using multiple linear regression, and total IgE levels were a continuous variable. Total IgE levels exceeding 150 kU/L were considered sensitized. Binary logistic regression analyses were employed to assess the correlation between metal exposure and the occurrence of an allergic state. Then, the association between co-exposure to the 11 metals and total IgE levels or the occurrence of sensitization status was further analyzed by Bayesian kernel machine regression (BKMR), a multi-contaminant model. There were 1429 adults with complete data included. Based on the median concentration, molybdenum (Mo) had the highest concentration (46.60 μg/L), followed by cesium (Cs), barium (Ba), lead (Pb), and mercury (Hg). And the median (interquartile range) for total IgE levels was 43.7 (17.3, 126.0) kU/L. Multiple linear regression results showed that Pb was significantly and positively associated with total IgE levels (β = 0.165; 95% CI: 0.046, 0.284). Binary logistic regression showed a significant positive correlation between urinary Pb (OR: 1.258; 95% CI: 1.052, 1.510) and tungsten (W) (OR: 1.251; 95% CI: 1.082, 1.447). Importantly, the BKMR model found a positive correlation between combined-metal exposure and total IgE levels and the occurrence of sensitization status. The mixed heavy-metal exposure was associated with increased total IgE levels, and this association may be driven primarily by the exposure of Pb and W. This study provides new insights into the relationship between heavy-metal exposure and allergic diseases. More research is needed to confirm these findings.

## 1. Introduction

Immunoglobulin E (IgE) is a key component of the network of proteins involved in the production of signaling responses to allergens or antigens [1]. Studies have revealed that elevated total IgE is a crucial feature of several allergic disease [2]. IgE mediates multiple allergic diseases, including allergic rhinitis (AR) and allergic asthma. The prevalence of IgE-mediated allergic diseases is becoming more prevalent every year and now accounts for 10% of children with food allergies [3] and 40% of the population with AR [4,5]. More than 330 million people worldwide suffer from asthma [6]. Among the atopic dermatitis (AD) population, the prevalence is approximately 20% in children and 2–18% in adults [7]. When the total IgE level in the body exceeds 150 kU/L, the organism is considered to be in a sensitized state [8]. Therefore, total IgE has been widely used as a biomarker for allergic diseases.

In industrialized societies, the incidence of allergic diseases, such as AD and asthma, has risen alarmingly over the past few decades [9]. Metal dust can cause the respiratory system to be sensitized, which can lead to respiratory diseases [10]. Around the world, the environment is heavily polluted by heavy metals, and soil and aquatic organisms are being adversely affected, causing serious impacts on human beings. Heavy metals persist long after they enter the ecosystem and are difficult to remove. The release of heavy-metal-rich industrial wastes can seriously contaminate soil, water, and air [11]. Importantly, studies have shown that metal dust is an environmental risk factor for a variety of allergic diseases (asthma, contact dermatitis, and hypersensitivity pneumonitis) [12,13,14]. Chromium (Cr), cobalt (Co) and Co compounds have been reported as asthma-causing substances [15], and a list of asthma-causing agents containing 25 metals has been established [16]. One study has mentioned a positive correlation between the prevalence of asthma and Cr, copper (Cu), selenium (Se), molybdenum (Mo), cadmium (Cd), and uranium (U) in urine among adults in a city in China [14]. Co-exposure to multiple metals (Cd, Hg, Pb, arsenic) has been positively correlated with asthma exacerbation [17]. Environmental lead (Pb) exposure has also been reported to correlate with elevated levels of IgE, and 38% of the total effects of Pb exposure on asthma were mediated by IgE levels [18,19]. It has also been noted that exposure to metals is associated with total IgE levels in adults [20].

The frequent presence of metals in the environment causes the human body to be exposed to multiple metals simultaneously. These metals can act on the body through synergistic or antagonistic effects [21,22]. In addition, when humans are simultaneously exposed to heavy metals, a single metal may also interact differently with other metals at different levels [23]. Single-pollutant models have limitations and drawbacks, such as the inability of the model to determine interactions between multiple metals, covariance, and nonlinear problems [24,25]. The novel Bayesian kernel machine regression (BKMR) methodology, which models the combined effects of mixture components, allowing for potentially correlated interactions and nonlinear effects, has attracted much attention in estimating health effects following exposure to mixtures [26]. The BKMR model can assess the overall effects of multiple pollutants on health indicators (total IgE levels) and further assess the interactions and nonlinear effects of different metals [27]. Therefore, this study aims to comprehensively evaluate the single- and combined-exposure effect relationships between total IgE and 11 urinary heavy-metal (Cd, Pb, Hg, Ba, Co, Cs, Mo, Sb, Tl, W, U) concentrations in adults and to identify potentially toxic heavy metals using multiple statistical models.

## 2. Methods

### 2.1. Participants

**Figure 1:**
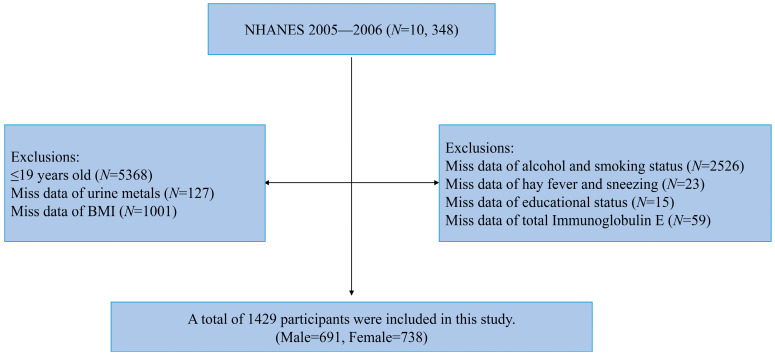
Flow chart for participants recruitment of the study.

The data for this study are based on the 2005–2006 National Health and Nutrition Examination Survey (NHANES), which was used to assess the health and nutritional status of adults in the United States. We selected this round of NHANES survey results for this study because only the 2005–2006 data contained information about total IgE. As shown in Figure 1, a total of 10,348 participants took part in this round of the NHANES survey. Next, we excluded people under 20 years of age, and then sequentially excluded people with missing data on urine metals, total IgE levels, and other relevant variables. Finally, 1429 participants were included—691 males and 738 females. The NHANES study protocol was approved by the National Health Statistics Research Ethics Review Board, and all participants signed an informed consent form (https://www.cdc.gov/nchs/nhanes/index.htm, accessed on 11 January 2023).

### 2.2. Urinary Heavy Metals Measurements

Urine samples were collected by trained nurses, processed, stored at −20 °C by trained full-time staff, and transported to the laboratory for further analysis, following quality control procedures. Inductively coupled plasma-mass spectrometry (ICP-MS), a multi-element analytical technique, was used to measure the following 10 elements: Co, Mo, Cd, antimony (Sb), cesium (Cs), barium (Ba), tungsten (W), thallium (Tl), Pb and uranium (U). Moreover, urine Hg concentrations were determined by Inductively coupled plasma-dynamic reaction cell-mass spectroscopy (ICP-DRC-MS). Before testing, the diluent was first prepared as a mixture of 2% (*v*/*v*) double-distilled concentrated nitric acid (GFS Chemicals Inc. Columbus, OH, USA, www.gfschemicals.com) and 1.5% (*v*/*v*) ethanol (Pharmco Products). Subsequently, internal standards of iridium (Ir), rhodium (Rh) and gallium (Ga) were added for multi-internal standardization, and the concentration of the three internal standards was guaranteed to be 10 μg/L. The urine sample was then diluted 10 times with this mixture. Detailed descriptions and procedures are shown on the NHANES website (https://wwwn.cdc.gov/Nchs/Nhanes/2005-2006/UHM_D.htm, accessed on 11 January 2023). In this study, we selected 11 metals with detection rates greater than 80%, meaning that the concentration of each metal exceeded the limit of detection in >80% of the samples, including Co, Mo, Cd, Sb, Cs, Ba, W, Tl, Pb, U, and Hg (Table S1). Values below the limits of detection (LOD) were estimated by dividing the LOD by the square root of two.

### 2.3. Serum Total IgE Measurements

Blood samples were collected, processed, and stored by trained staff and transported to the laboratory for further analysis. Serum total IgE was detected using the Pharmacia Diagnostics ImmunoCAP 1000 System (Kalamazoo, MI, USA). Anti-IgE covalently coupled to the ImmunoCap™ reaction vessel can react with total IgE in the sample. A detailed description of the laboratory method used can be found on the NHANES website (https://wwwn.cdc.gov/Nchs/Nhanes/2005-2006/AL_IGE_D.htm, accessed on 11 January 2023). Briefly, after washing, the enzyme-labeled anti-IgE antibody was added to form a complex. After incubation, the unbound enzyme-anti-IgE was washed away, and the bound complex was then incubated with the developer. After stopping the reaction, the fluorescence of the eluate was measured. The fluorescence was proportional to the concentration of IgE antibody in the sample. For total serum IgE measurement, the LLOD (Lower Limit of Detection) was set at 2.00 kU/L. For results analyzed below the LLOD, the results were substituted with an estimate—the LLOD divided by the square root of two.

### 2.4. Covariates

Participants’ demographic details and lifestyles were assessed through a standardized questionnaire, including age (years), race (Mexican American, Other Hispanic, Non-Hispanic Black, Non-Hispanic White, and Other Race), gender, education level (above high school, high school, and below high school), BMI categories (<25 kg/m^2^, 25–30 kg/m^2^, ≥30 kg/m^2^) [28], smoking status, drinking status, and annual household income. Annual household income included low income (<20,000 dollars) and high income (≥20,000 dollars). Alcohol consumption included both drinkers (≥12 drinks/year) and non-drinkers (<12 drinks/year). Smokers were defined as “having smoked at least 100 cigarettes in their lifetime”.

### 2.5. Statistical Analysis

Descriptive statistics analyzed demographic characteristics. The categorical variables were presented as proportions. Normal data were expressed as mean ± standard deviation (*SD*), while non-normal data were described using median (quartiles). The Mann–Whitney test was used to compare metal concentrations between groups based on whether they were in a sensitized state. Urinary heavy-metal concentrations were ln-transformed to conform to a normal distribution before proceeding to the next step of statistical analysis. Spearman‘s analysis was used in this study to estimate the correlation between metals and total IgE. In order to estimate the effect of exposure to heavy-metal mixtures on total IgE levels in humans more accurately, three statistical models, namely multiple linear regression, multiple logistic regression, and BKMR, were used for comprehensive analysis.

(1) The concentration of each metal was creatinine-corrected and ln-transformed, which was first performed with multiple linear regression. In the crude model, we included each of the 11 metals as an independent variable and total IgE as the dependent variable. In the adjusted model, multiple covariates (age, gender, race/ethnicity, educational level, drinking status, smoking status, BMI, and annual household income) were also included.

(2) In logistic regression models, we observed the links between multiple metal exposure levels and the occurrence of sensitized states. Total IgE was entered into the model as the dependent variable and heavy-metal concentration was the independent variable, respectively. Moreover, consistent with multiple linear regression, we used two models (the crude model and the adjusted model).

Due to the correlations and interactions of the components in heavy-metal mixtures, the relationship between mixtures and health would be underestimated using traditional single-metal exposure models [29], so we used a new mixture exposure model for our analyses next.

(3) Bayesian kernel machine regression (BKMR) provides flexible and concise estimations of exposure-response functions containing nonlinear and non-additive effects; fits models with high variable correlation well; and adjusts for a wide range of covariates, in addition to identifying significant components of mixtures [30]. This study used posterior inclusion probabilities (PIPs) analysis to estimate the significance of the effect of the metals on total IgE changes. The univariate dose-response curve for a single metal versus a change in total IgE can be plotted with the other metals fixed at the 50th percentile. By fixing the concentrations of the remaining metals at percentile P25, P50, or P75, the individual exposure effects of each metal on total IgE levels could be determined. In addition, this study assessed the interaction of heavy metals with total IgE changes and also estimated the overall effect of heavy-metal mixtures. We set a component-wise variable selection with 10,000 iterations using a Markov Chain Monte Carlo (MCMC) algorithm and set the seed at 10 when fitting the hierarchical BKMR model to repeat the results.

Data analysis was performed using R (Version 4.2.0, R Foundation for Statistical Computing, Vienna, Austria). Two-sided *p*-values less than 0.05 were considered statistically significant.

## 3. Results

### 3.1. Characteristics of the Participants

In this study, 1429 participants were included, and their demographic characteristics are presented in Table 1. In this study, 698 (48.5%) participants were men and 742 (51.5%) were women. Most participants were 20–45 years old (47.4%), non-Hispanic white (51.8%), had more than high school education (48.2%) and had an annual household income of USD 20,000 or more (76.9%). There were 688 (47.8%) smokers and 988 (67.9%) drinkers. The median (interquartile range) of total IgE level was 43.8 (17.30, 127.0) kU/L, and the median (interquartile range) urinary creatinine level of the participants was 119.0 (70.0, 174.0) mg/dL.

### 3.2. Urine Metal Concentration Distribution and Correlation

**Figure 2:**
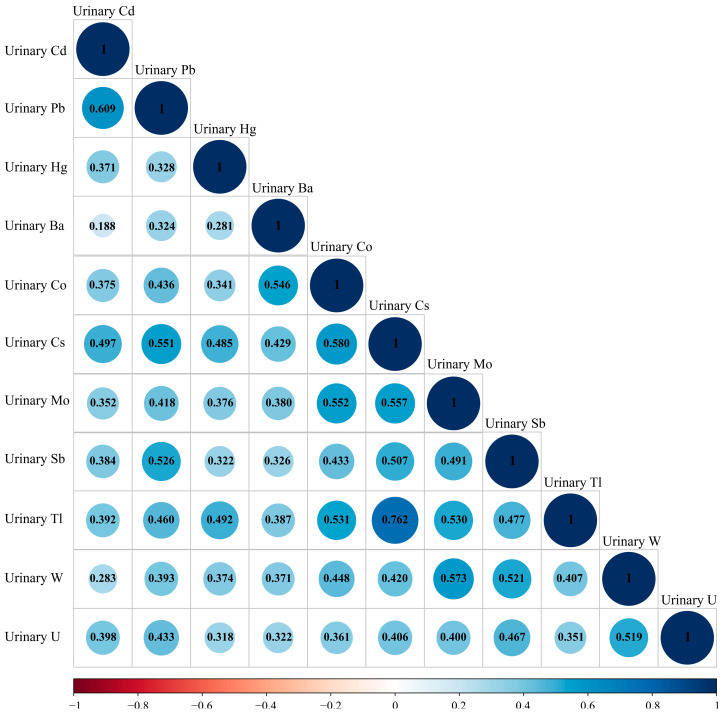
Spearman correlations among the mixed exposure of eleven metals in the participants.

The 11 metals (Cd, Pb, Hg, Co, Cs, Ba, Mo, Sb, Tl, W, U) with a detection rate greater than 80% were included in this study. The distribution of metal concentrations in urine is shown in Table 2. Based on the median concentration, Mo had the highest concentration (46.60 g/L), followed by Cs (5.06 g/L), Ba (1.31 g/L), Pb (0.66 g/L), and Hg (0.46 g/L). Figure 2 shows a plot of the correlation coefficients for pairs of metals calculated by Spearman’s rank correlation analysis, showing the correlation between the metals. The results indicated that almost all metals exhibited a positive correlation with *p*-values less than 0.05, and the correlation coefficients ranged from 0.188 to 0.762. The most significant correlation was observed between Cs and Tl (*r* = 0.762). The correlation coefficients for Pb and Cd, Co and Cs, Mo and W, Cs and Mo, Co and Mo, Pb and Cs, Ba and Co, Co and Tl, Mo and Tl, Pb and Sb, Sb and W, and W and U were 0.609, 0.580, 0.573, 0.557, 0.552, 0.551, 0.546, 0.531, 0.530, 0.526, 0.521, and 0.519, respectively. These results show that there might be co-linearity between multiple metals. Therefore, we proceeded to assess the impact of metal mixtures on total IgE using multi-contaminant exposure models.

### 3.3. Association between Heavy-Metal Exposure and Total IgE Using Multiple Linear Regression

**Figure 3:**
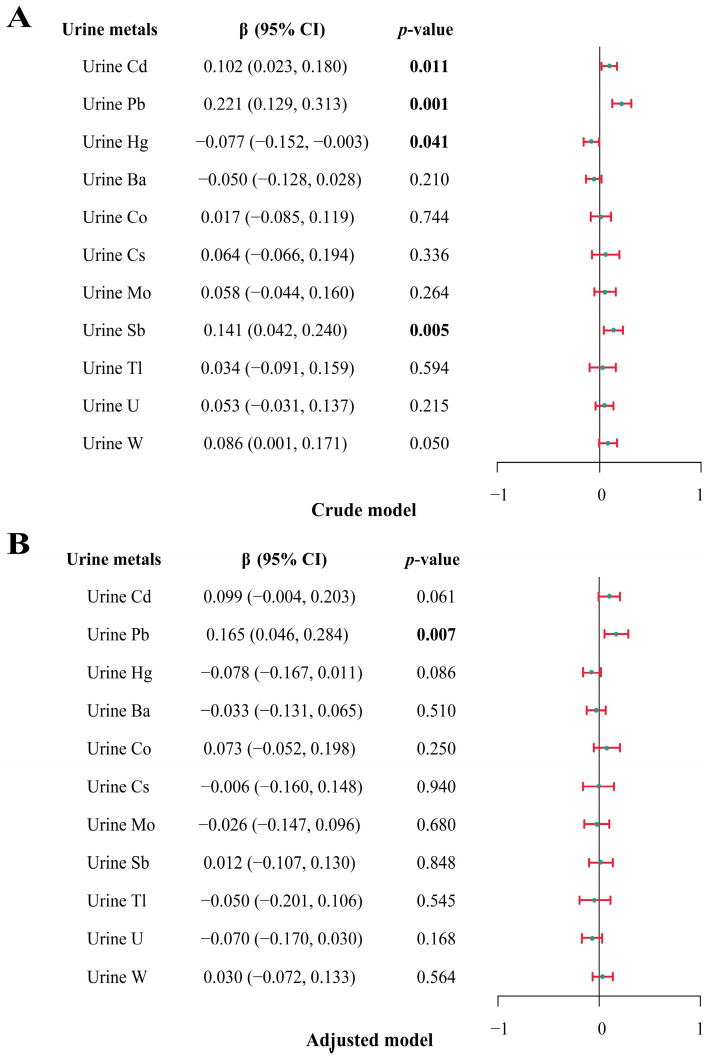
Association between urinary metal concentrations (ln-transformed) and total IgE by multivariate linear regression (*n* = 1429). (**A**). Crude model: not adjust for covariates. (**B**). Adjusted model: variables of age, gender, race/ethnicity, educational level, drinking status, smoking status, BMI, and annual household income were adjusted. Bold numbers indicated *p* < 0.05.

The correlation results showed a significant positive correlation between total IgE and various metals, including Cd, Pb, Sb, and W (Figure S1). Figure 3 demonstrates the relationship between various metals and total IgE found using multiple linear regression modeling. The results showed a strong positive correlation between total IgE and Pb (crude model: β = 0.222; 95% CI: 0.130, 0.314; adjusted model: β = 0.160; 95% CI: 0.041, 0.279). Furthermore, we also found that Cd (β = 0.103; 95% CI: 0.024, 0.181) and Sb (β = 0.147; 95% CI: 0.048, 0.245) were significantly and positively correlated with total IgE, while Hg (β = −0.080; 95% CI: −0.152, −0.008) was significantly and negatively correlated with total IgE.

### 3.4. Multiple Logistic Regression Analysis of the Relationship between Multiple Metals and Total IgE

**Figure 4:**
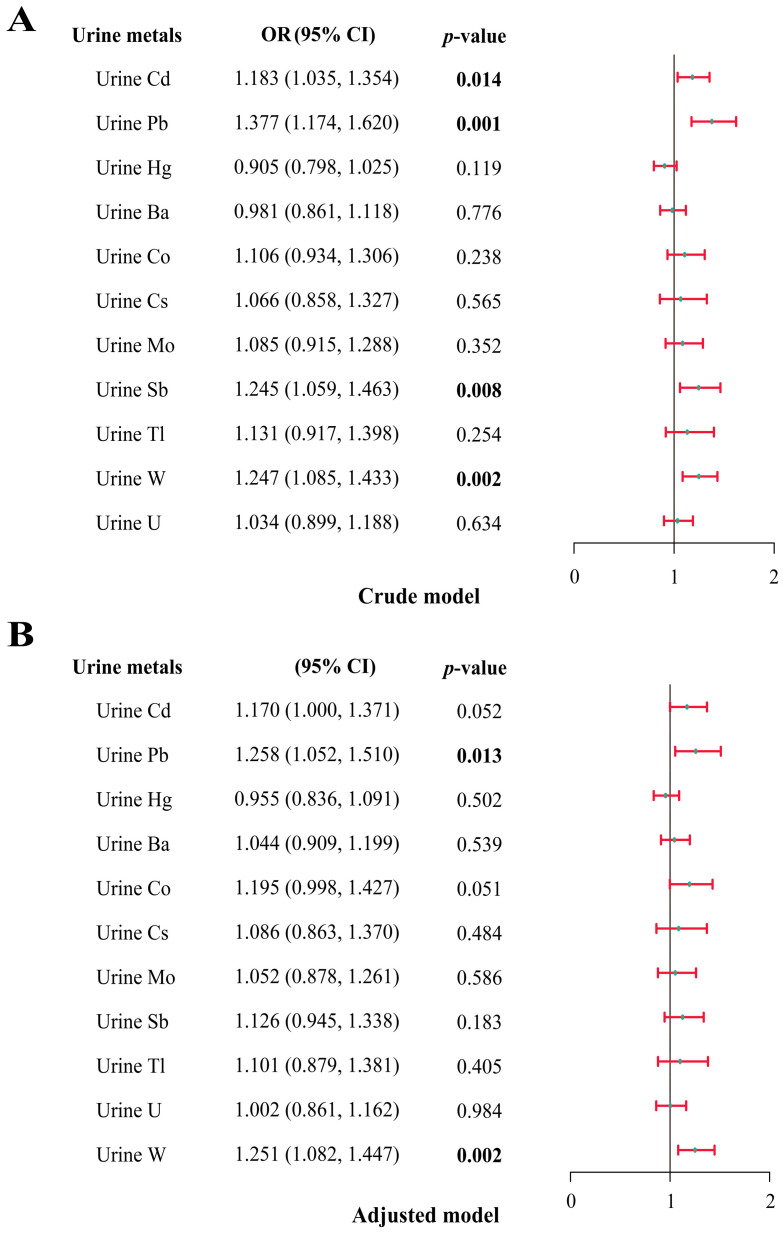
Association between urinary metal concentrations (ln-transformed) and total IgE by logistic regression (*n* = 1429). (**A**). Crude model: not adjust for covariates. (**B**). Adjusted model: variables of age, gender, race/ethnicity, educational level, drinking status, smoking status, BMI, and annual household income were adjusted. Bold numbers indicated *p* < 0.05.

Next, total IgE was transformed into a categorical variable with a threshold of 150 kU/L, representing the onset of allergic status. We found significantly higher concentrations of several metals, including Cd, Pb, Sb, and W, in populations with elevated total IgE. (Figure S2). The relationship between urinary metals and allergic status was investigated by multiple logistic regression (Figure 4). In the adjusted model, the risk of total IgE elevation increased by 25.8% (OR: 1.258; 95% CI: 1.052, 1.510) and 25.1% (OR: 1.251; 95% CI: 1.082, 1.447) for each one-unit increase in Pb and W concentrations, respectively. Meanwhile, in the crude model, we observed that Cd, Pb, Sb, and W were positively correlated with allergic status. To further determine the effect of metals on allergy status, we examined the association between metals and whether participants had been told by a doctor that they had an allergy (Table S2). We similarly observed a significant positive association between W and participants who had been told by a doctor that they had an allergy (crude model: OR = 1.141; 95% CI: 1.007, 1.292; adjusted model: OR = 1.170; 95% CI: 1.025, 1.337).

### 3.5. Effects of Exposure to Multiple Metals on Total IgE Using the BKMR Model

Subsequently, the nonlinear, interaction, and cumulative effects of the 11 metals on total IgE were evaluated in the BKMR model using total IgE as a continuous and categorical variable, respectively. Table 3 summarizes the posterior inclusion probabilities (PIPs), which can be seen as an indicator of relative importance in mixed exposure, with closer to 1 representing greater importance and closer to 0 representing less importance for the BKMR model. The PIP value for Pb was 0.843 when total IgE was a continuous variable and 0.773 when total IgE was a categorical variable, suggesting that the contribution of Pb was the largest in both mixed-exposure models and dominated the overall effect. We observed a positive correlation between urinary metal concentrations and total IgE levels and a monotonous upward trend in the overall effect curve.

**Figure 5:**
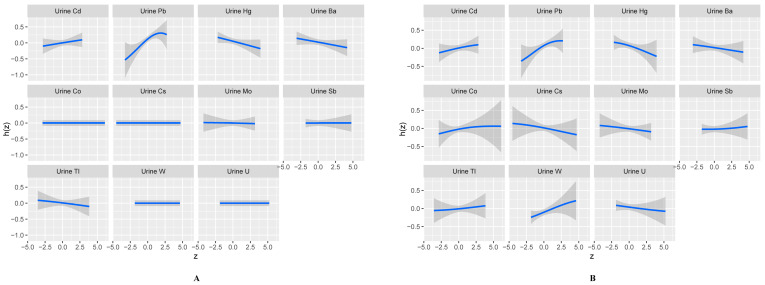
Univariate exposure-response functions and a 95% confidence interval for each metal exposure fixed at the median. The total IgE was continuous variables (**A**) and categorical variables (**B**).

Figure 5 shows the univariate exposure-response functions for each metal in urine with respect to total IgE. When total IgE was a continuous variable (Figure 5A), there was a notable positive correlation between total IgE and Cd and Pb and a negative correlation between total IgE and Hg, Ba, and Tl. When total IgE was the categorical variable (Figure 5B), we also observed a positive correlation between the occurrence of allergic states and Cd and Pb and a negative correlation between the occurrence of allergic states and Hg and Ba. Positive correlations were also observed between the occurrence of allergic states and Co and W, and negative correlations were found with Cs, Mo, and U.

**Figure 6:**
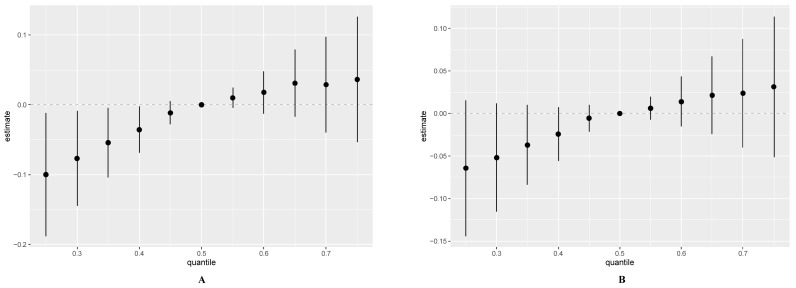
Associations between the overall metal mixture and total IgE by Bayesian kernel machine regression analysis. The total IgE was continuous variables (**A**) and categorical variables (**B**).

Figure 6 shows the cumulative effect of urinary metals when all elements were fixed to the median value. Notably, we observed that, likewise, metal-mixing exposure was positively associated with participants who had been told they had an allergy (Figure S3). We looked at potential pairwise interactions by plotting binary exposure-response functions (Figure S4), and we did not observe interactions between all metals and total IgE levels when total IgE was a continuous variable (Figure S4A). When total IgE was the categorical variable (Figure S4B), the interaction between the exposure factors was not significant, except for Cd and Pb. As shown in the figure, the slope of the positive correlation between lead and total IgE flattened as the mercury concentration increased. However, its exact relationship requires further mechanistic studies.

**Figure 7:**
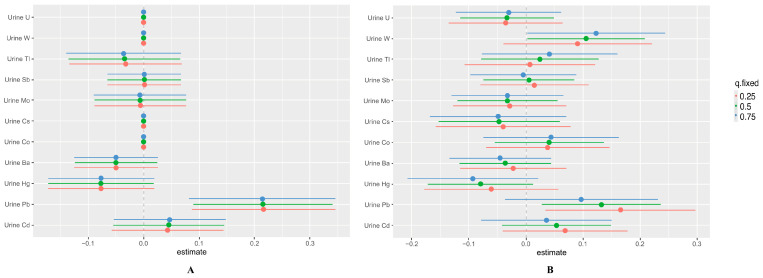
Single-exposure effects of individual metals on total IgE (estimates and 95% confidence intervals) when all other metals were fixed at their 25th, 50th, or 75th percentile. The total IgE was continuous variables (**A**) and categorical variables (**B**).

To fully explore potential interactions with metal mixtures, Figure 7 analyzes the single-exposure effects (estimates and 95% confidence intervals) of individual metals on total IgE levels when all other elements were fixed at different quartiles (25th, 50th, and 75th). When total IgE was a continuous variable and other metals were set at the 25th, 50th, and 75th quartiles, Pb was significantly and positively associated with total IgE levels (Figure 7A). When total IgE was a categorical variable and other metals were set at the 50th and 75th quartiles, W was significantly and positively correlated with the occurrence of allergic states, and when other metals were set at the 25th and 50th quartiles, Pb was positively correlated with the occurrence of allergic states (Figure 7B).

## 4. Discussion

In this study, we identified the metals associated with the development of allergic states using multiple linear regression and binary logistic regression and further assessed the specific effects of mixed-metal exposure on allergic states using the BKMR model. We found that urinary Pb and urinary W were risk factors for elevated total IgE and the occurrence of allergic states. In the BKMR model, we found agreement between the single-exposure effects model and the multi-pollutant effects analysis. The BKMR model also suggested that urinary Cd and W exposure might be important contributors to elevated total IgE. In conclusion, we found that urinary Pb and urinary W might have a critical role in the positive correlation between elevated total IgE and the occurrence of allergic states.

There have been a number of studies looking at the specific mechanisms by which metal exposure leads to elevated IgE levels or allergic disease, but there are still a large number of unknown mechanisms that need to be explored. Pb has been classified as one of the major environmental pollutants causing public health problems [31]. Total IgE levels were found to be positively correlated with blood lead levels in 4979 participants aged 20 years and older [32]. In a cross-sectional study involving 930 children in Taiwan, China, blood Pb concentration was positively associated with total IgE levels [19]. Similar to previous studies, we have identified a positive correlation between urinary Pb and both total IgE levels and the development of allergic status [18,19]. Recently, it has been suggested that increased blood Pb concentrations were associated with the accelerated synthesis of IgE and pro-inflammatory biomarkers, and a higher risk of allergies, asthma, and atopic diseases in children exposed to a variety of high concentrations of heavy metals has also been mentioned [33,34].

The mechanism by which Pb exposure is associated with elevated total IgE levels is not fully understood. However, Pb exposure may contribute to the development of hypersensitivity. It has been reported that the exposure of pregnant women to the heavy metal Pb during the prenatal or early postnatal period skews the immune response in favor of Th2 and promotes the production of IgE or Th2-related cytokines. It has also been shown in animal studies that the exposure of mice to metal (Pb) promotes Th2 responses and inhibits IFN-γ expression, which disrupts the balance between Th1 and Th2, decreases the Th1-Th2 ratio, and results in the production of large amounts of Th2 cytokines (such as IL-4, IL-5, etc.). The study also noted that exposure to metal (Pb) damages natural killer cells that produce IFN-c, which can inhibit IgE production [35,36]. Therefore, in combination with the results of this study, we suggest that Pb may be an important environmental risk factor for allergic diseases.

Next, we similarly found that W may be an important risk factor for allergic diseases. W is a unique transition metal with several desirable properties, including strength, flexibility, a high melting point, and good electrical conductivity [37]. As a result, W is widely used in a variety of industrial products [38], which, at the same time, inevitably leads to an increase in the number of people exposed to high concentrations of W. It has been reported that exposure to the metal W may be associated with the production of autoimmune biomarkers [39]; in addition, it has been reported that W (including tungsten carbide) is immunotoxic to the mature and developing immune system in both in vitro and in vivo models. However, no studies have been published on populations exposed to high levels of W to show that W has a direct immunotoxic effect, and more cohort studies and experimental studies are needed to provide evidence. W may simultaneously enhance the effects of other co-exposure factors, leading to greater toxicity (for example, a tungsten carbide-cobalt mixture has an additive effect on cell death)_;_ this finding suggests how W toxicity should be assessed in the future and may explain why many previous studies have failed to find W toxicity [37]. Therefore, although the exact mechanism of toxicity of metal W in causing elevated total IgE levels cannot be accurately concluded, based on the results of now-published studies and the present study, we believe that metal W may affect total IgE production in vivo and influence the development of allergic diseases in humans. However, we still need more animal or cellular experiments to further explore the mechanism of toxicity.

Of note, our results show that urinary Hg concentrations showed a negative correlation with the occurrence of sensitized states, which is inconsistent with the results of the existing literature, possibly because mixed-metal interactions may alter toxicokinetics and complicate studies. Through BKMR modeling, this study found that there was an interaction between Pb and Hg. Figure 7B shows that the relationship between elevated mercury exposure and changes in outcomes was not significant when Pb concentrations were at the 75th percentile, whereas the correlation between elevated Hg exposure and elevated outcomes was stronger when Pb was at the 25th percentile. Therefore, the development of a new hybrid model may be beneficial to simulate real exposures in the environment and reveal the true association. Additionally, it has been mentioned that exposure to HgCl_2_ can also promote the high production of Th2-related cytokines and aggravate allergic diseases [40], but in the present study, the concentration of Hg exposure in the population showed a weak negative correlation with total IgE, so we hypothesized that it is possible for Hg to reduce the production of total IgE at low exposure. Furthermore, it is also possible that it could be due to the different ways of collecting the samples and the type of population included in the study. However, the available data completeness, sample size, and relevant evidence are not sufficient, and we need to be cautious about extrapolating these results.

In addition, in all models, we observed that urinary Cd showed some positive correlations with elevated total IgE levels, and although the differences were not statistically significant in the linear and logistic regression models with the inclusion of covariates, the health risk continues to warrant our attention, and its toxicological mechanisms leading to elevated total IgE may be via multiple pathways.

Currently, the increased exposure of humans to metal mixtures is an obvious environmental problem. In this study, we were not only satisfied with caring about the effect of a single metal on total IgE levels; we also used a different approach to find associations between mixed-metal exposures and the occurrence of allergic states or IgE levels, which helped us to better understand the relationship between metals and allergic diseases. The application of new algorithms and models can circumvent some of the shortcomings of traditional statistical models, such as the inability to determine interactions between multiple metals, covariances, and nonlinearities [24,25]. BKMR can help assess the comprehensive effect of all metallic elements on the occurrence of allergic states and also investigate the potential nonlinearities and interactions of multiple metal exposures. We also adjusted for some important potential confounding factors. Firstly, we analyzed the association of single metals with total IgE levels using linear regression and logistic regression models, and from both models, we observed a positive correlation between urinary Pb, urinary W, and elevated total IgE. Secondly, by looking at the results from the BKMR model, it can be seen that total IgE levels increase with increasing concentrations of Pb and W exposure, and in addition, it can be concluded that mixed exposures to multiple metals are positively correlated with increased total IgE levels. In this study, we obtained the same results from different models, which proves the correctness of the conclusions. It is worth noting that the accounting approach of the BKMR model may be a limitation [41]. The BKMR model, which fixes other chemicals at a certain level in order to extrapolate the exposure-response function, is unable to estimate the effects of co-exposure patterns with both high and low levels of chemicals.

The present study has some inherent limitations. Firstly, this study was only a cross-sectional investigation and therefore we are unable to determine a cause-and-effect relationship between urinary metal exposure and total IgE levels. Thus, more prospective cohort studies and experimental studies to provide relevant evidence are required. Second, urinary metal levels are biomarkers of recent exposure and may not fully reflect long-term or cumulative exposure. The effects of metals on total IgE and allergic reactions need to be further explored in combination with more sensitive internal (e.g., blood, hair, bone) and external exposure levels in the future. Finally, the study used only 2005–2006 data for United States adults, so the sample size was small, limiting our interpretation of the results and making extrapolation to children, adolescents, or other geographic populations difficult. There is also the possibility of survey data bias or unmeasured factors.

Therefore, the present study has identified possible environmental risk factors (Pb and W) for allergic diseases based on single-pollutant exposure models and multi-pollutant exposure models, as well as positive associations between mixed-metal exposures and allergic diseases, which is important for future mechanistic studies of allergic diseases. In the future, we hope that we can further combine molecular biology studies to determine the specific roles of these environmental risk factors and their mixed exposures in different allergic diseases, such as allergic rhinitis, through animal and cell experiments. Moreover, heavy-metal exposure may cause a wide range of diseases, including allergic diseases, and the risk increases exponentially over time when people are exposed to heavy metals from multiple sources, even in small amounts. People should minimize their exposure to heavy metals—for example, by using healthy and environmentally friendly household products and establishing a healthy lifestyle—to help prevent and reduce the occurrence of diseases caused by heavy-metal exposure.

## 5. Conclusions

In summary, in this cross-sectional study of 1429 United States adults, we observed that some metals such as Pb and W could affect total IgE levels and the occurrence of sensitized states. A nonlinear association was observed between metals and total IgE levels or the development of sensitized states. Thus, the results suggest a complex relationship between multi-metal exposure and the onset of sensitization. Subsequent studies would be necessary to confirm our results and explore the specific mechanisms involved, to provide a basis for precise protection, exposure reduction, and treatment.

## Figures and Tables

**Table 1:** Characteristics of participants included in the study, NHANES 2005–2006 (*n* = 1429).

Variable	Catalogue	*N* (%) or Median [IQR]
Age (years old)	20–45 years old	674 (47.2)
	46–65 years old	429 (30.0)
	>65 years old	326 (22.8)
Gender	Male	691 (48.4)
	Female	738 (51.6)
Race/ethnicity	Mexican American	277 (19.4)
	Other Hispanic	43 (3.0)
	Non-Hispanic White	742 (51.9)
	Non-Hispanic Black	320 (22.4)
	Other Race	47 (3.3)
Body mass index (kg/m^2^)	Normal	445 (31.1)
	Overweight	500 (35.0)
	Obesity	484 (33.9)
Educational level	Below than high school	402 (28.1)
	High school	325 (22.7)
	Above than high school	702 (49.1)
Income (USD)	<20,000	331 (23.2)
	≥20,000	1098 (76.8)
Smoke	No	746 (52.2)
	Yes	683 (47.8)
Alcohol	No	458 (32.1)
	Yes	971 (67.9)
Total Immunoglobulin E (kU/L) (median [IQR])		43.7 [17.3, 126.0] ^a^
Creatinine (mg/dL) (median [IQR])		119.0 [70.0, 174.0]

^a^: Median and inter-quartile range.

**Table 2:** Distribution of urine metal concentrations, NHANES 2005–2006 (*n* = 1429).

Metal	Detection Frequency	Percentile				
		5th	25th	50th	75th	95th
Cd	88.77	0.02	0.14	0.28	0.53	1.24
Pb	97.57	0.12	0.36	0.66	1.14	2.53
Hg	92.00	0.06	0.22	0.46	1.01	3.00
Ba	98.86	0.22	0.68	1.31	2.57	6.67
Co	99.61	0.10	0.23	0.37	0.58	1.45
Cs	100.00	1.27	3.07	5.06	7.55	12.40
Mo	100.00	8.68	25.70	46.60	74.95	148.15
Sb	84.64	0.02	0.04	0.07	0.12	0.32
Tl	99.88	0.04	0.10	0.17	0.25	0.41
W	91.86	0.02	0.04	0.08	0.14	0.43
U	92.31	0.00	0.00	0.01	0.01	0.03

**Table 3:** Posterior inclusion probabilities (PIPs) derived from the BKMR model.

Metal ^a^	PIPs	Metal ^b^	PIPs
Cd	0.352	Cd	0.008
Pb	0.854	Pb	0.837
Hg	0.453	Hg	0.006
Ba	0.230	Ba	0.003
Co	0.287	Co	0.000
Cs	0.271	Cs	0.000
Mo	0.178	Mo	0.003
Sb	0.209	Sb	0.002
Tl	0.154	Tl	0.010
U	0.159	U	0.000
W	0.486	W	0.000

^a^ The total IgE was continuous variables. ^b^ The total IgE was categorical variables.

## Data Availability

The data are publicly available on the NHANES website: https://www.cdc.gov/nchs/nhanes/index.htm, accessed on 11 January 2023.

## Supplementary Materials

The following supporting information can be downloaded at: https://www.mdpi.com/article/10.3390/toxics12020116/s1, Figure S1: The spearman correlation between urinary metal concentrations after log-transformation and total IgE levels after log-transformation; Figure S2: The comparison of metal concentrations between groups based on sensitisation or not; Figure S3: Associations between the overall metal mixture and whether participants had been told by a doctor that they had an allergy by Bayesian kernel machine regression analysis; Figure S4. The interaction of urine multiple metals on total IgE. The total IgE was continuous variables (A) and categorical variables (B); Table S1: Urinary metal detection rate; Table S2. Association between metals and whether participants had been told by a doctor that they had an allergy by logistic regression (n = 1424).
