# Supplementary material for: The Associations between Exposure to Multiple Heavy Metals and Total Immunoglobulin E in U.S. Adults
Source: Toxics. 2024 Jan 30;12(2):116. doi: 10.3390/toxics12020116 (PMC10891582; doi:10.3390/toxics12020116)
Supplement: Supplementary file 1 [file toxics-12-00116-s001.zip › toxics-2821609-supplementary.pdf]

Supplementary Materials: The Associations between Multiple Heavy Metals Exposure and Total Immunoglobulin E in U.S. Adults

Xin Song, Xiaowen Ding, Piye Niu, Tian Chen and Tenglong Yan

Table S1. Urinary metal detection rate.

Urinary Metal	Unit	LOD	Detection rate[a] (%)
Ba	μg/L	0.12	98.87
Cd	μg/L	0.042	89.91
Co	μg/L	0.041	99.61
Cs	μg/L	0.066	100
Mo	μg/L	0.92	100
Pb	μg/L	0.10	97.63
Sb	μg/L	0.032	86.68
Tl	μg/L	0.015	99.88
W	μg/L	0.021	92.47
U	μg/L	0.0017	92.86
Hg	μg/L	0.08	92.59

[a] Percentage of people with measured metal concentrations greater than the detection limit.

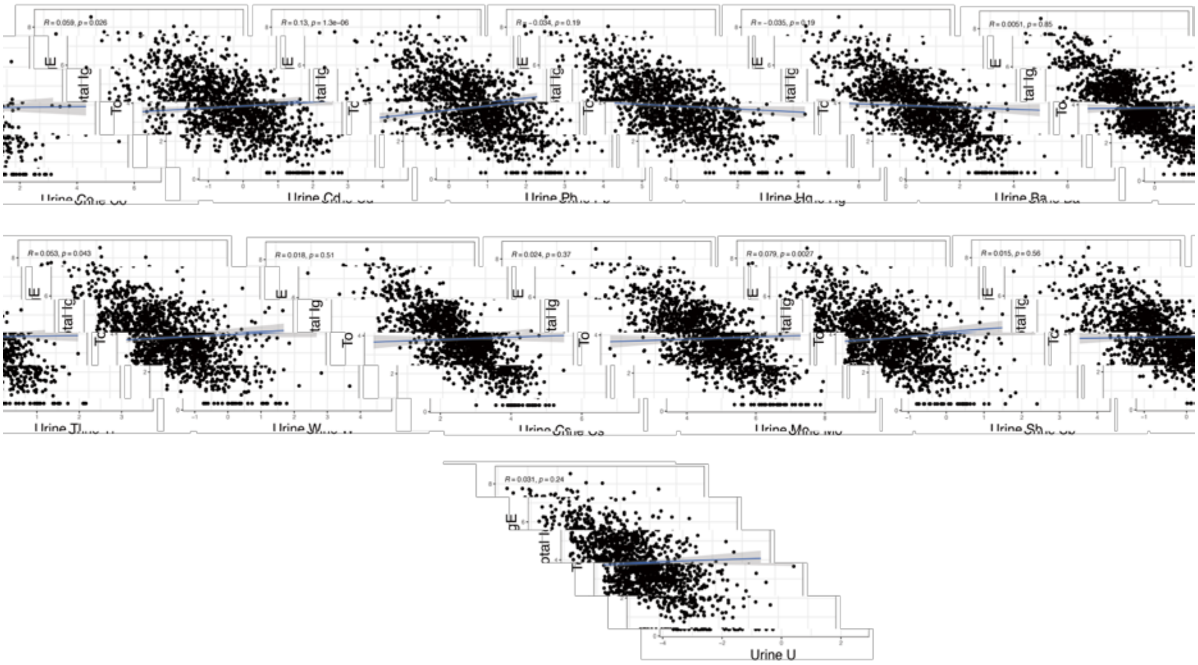

Figure S1. The spearman correlation between urinary metal concentrations after log-transformation and total IgE levels after log-transformation.

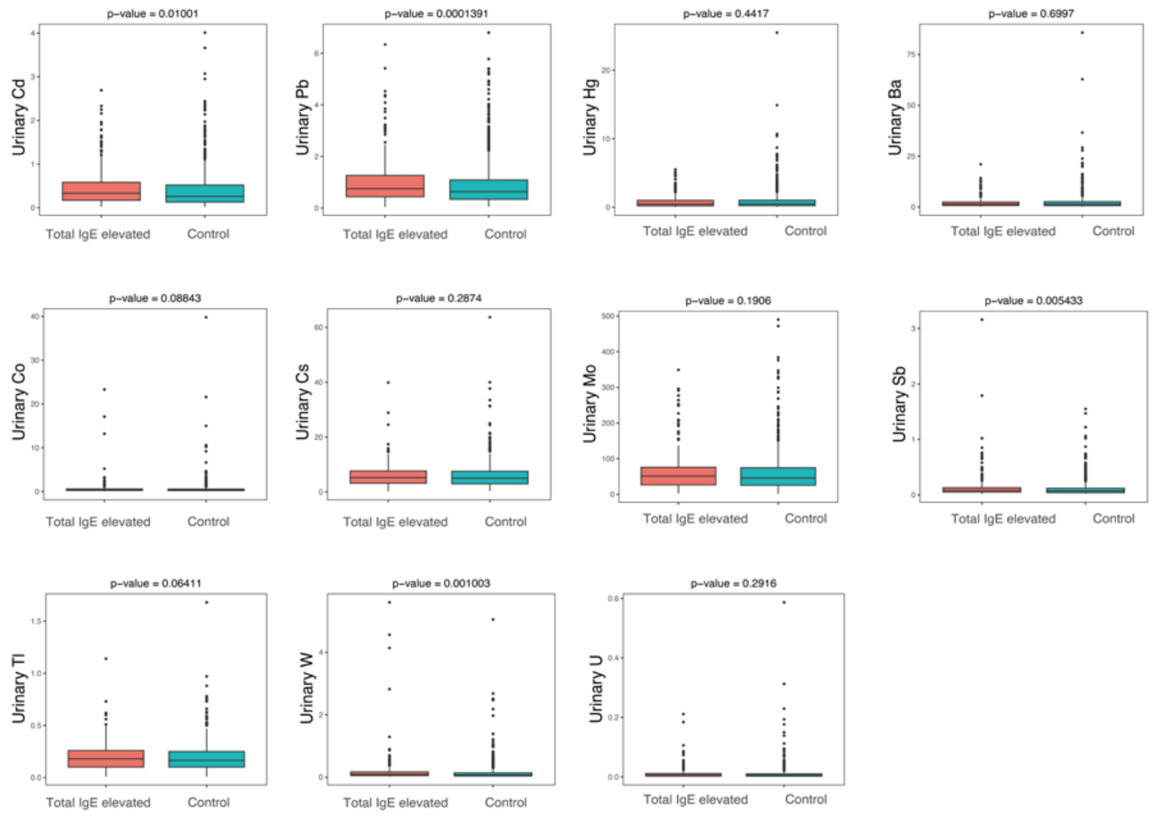

Figure S2. The comparison of metal concentrations between groups based on sensitisation or not.

Table S2. Association between metals and whether participants had been told by a doctor that they had an allergy by logistic regression (n = 1424).

	**Unadjusted**[1]		**Adjusted**[2]	
	**OR (95%CI)**	*p*	OR (95%CI)	*p*
Urinary Cd	1.037(0.924, 1.166)	0.538	1.116(0.976, 1,279)	0.110
Urinary Pb	0.837(0.730, 0.959)	0.011	1.007(0.861, 1.180)	0.926
Urinary Hg	1.191(1.067, 1.330)	0.002	1.113(0.990, 1.252)	0.072
Urinary Ba	1.087(0.969, 1.222)	0.153	0.981(0.865, 1.111)	0.761
Urinary Co	1.020(0.877, 1.185)	0.793	0.975(0.829, 1.143)	0.753
Urinary Cs	1.070(0.883, 1.298)	0.489	1.015(0.830, 1.242)	0.886
Urinary Mo	1.045(0.899, 1.215)	0.566	1.117(0.953, 1.309)	0.172
Urinary Sb	0.905(0.780, 1.048)	0.186	1.004(0.855, 1.176)	0.962
Urinary Tl	1.251(1.038, 1.511)	0.019	1.132(0.925, 1.386)	0.230
Urinary W	1.141(1.007, 1.292)	0.039	1.170(1.025, 1.337)	0.020
Urinary U	0.999(0.882, 1.130)	0.989	1.082(0.948, 1.235)	0.241

[1] Crude model: not adjust for covariates. [2] Adjusted model: variables of age, gender, race/ethnicity, educational level, drinking status, smoking status, BMI, and annual household income were adjusted.

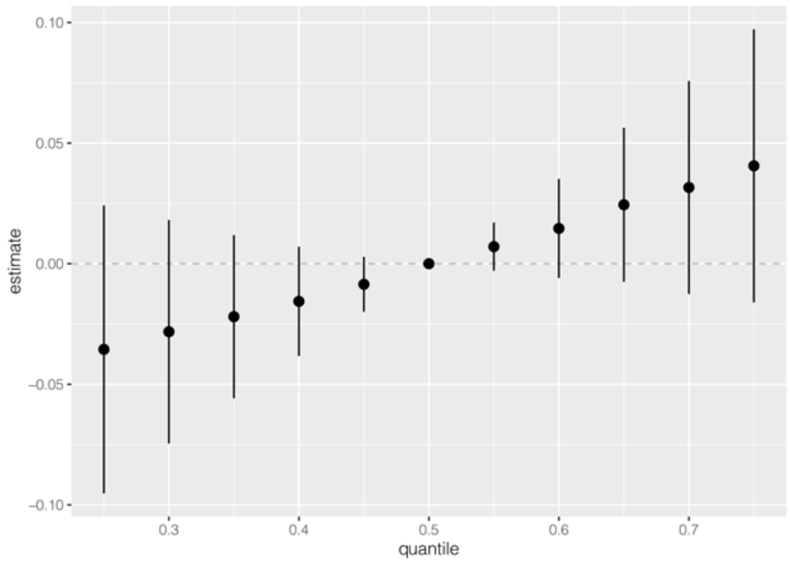

Figure S3. Associations between the overall metal mixture and whether participants had been told by a doctor that they had an allergy by Bayesian kernel machine regression analysis.

.

A

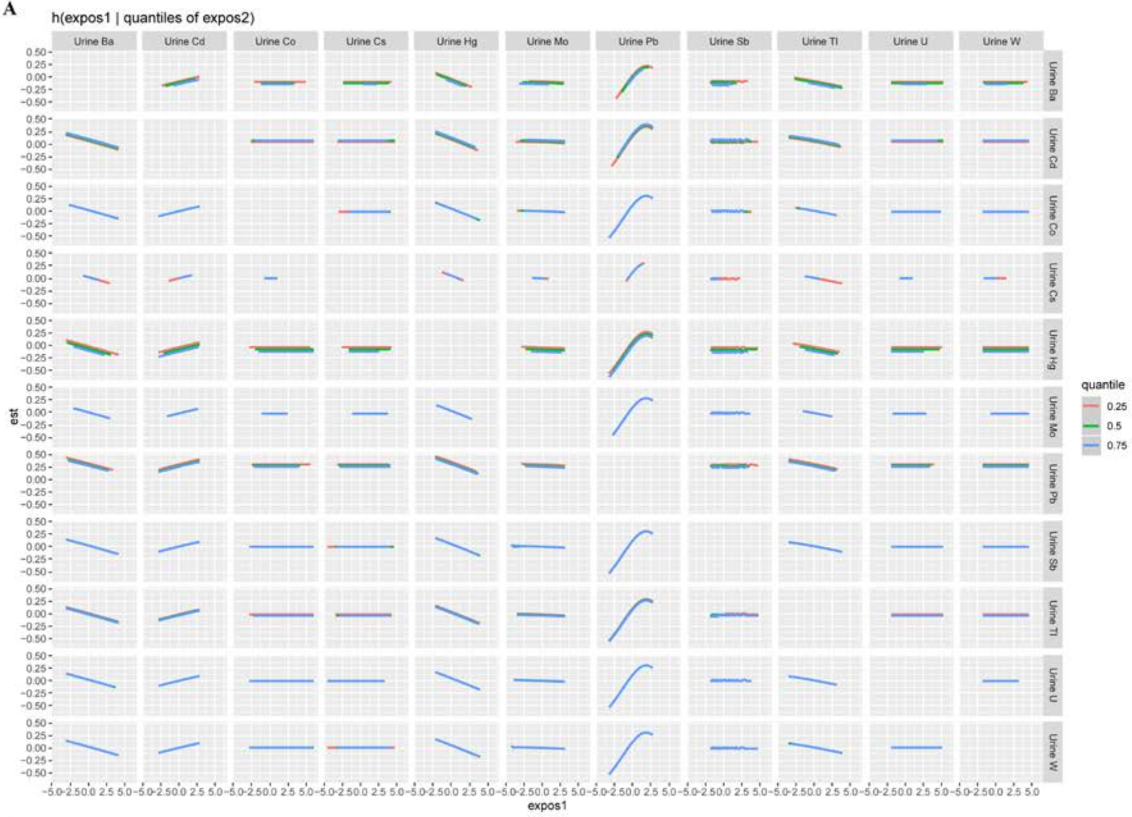

B

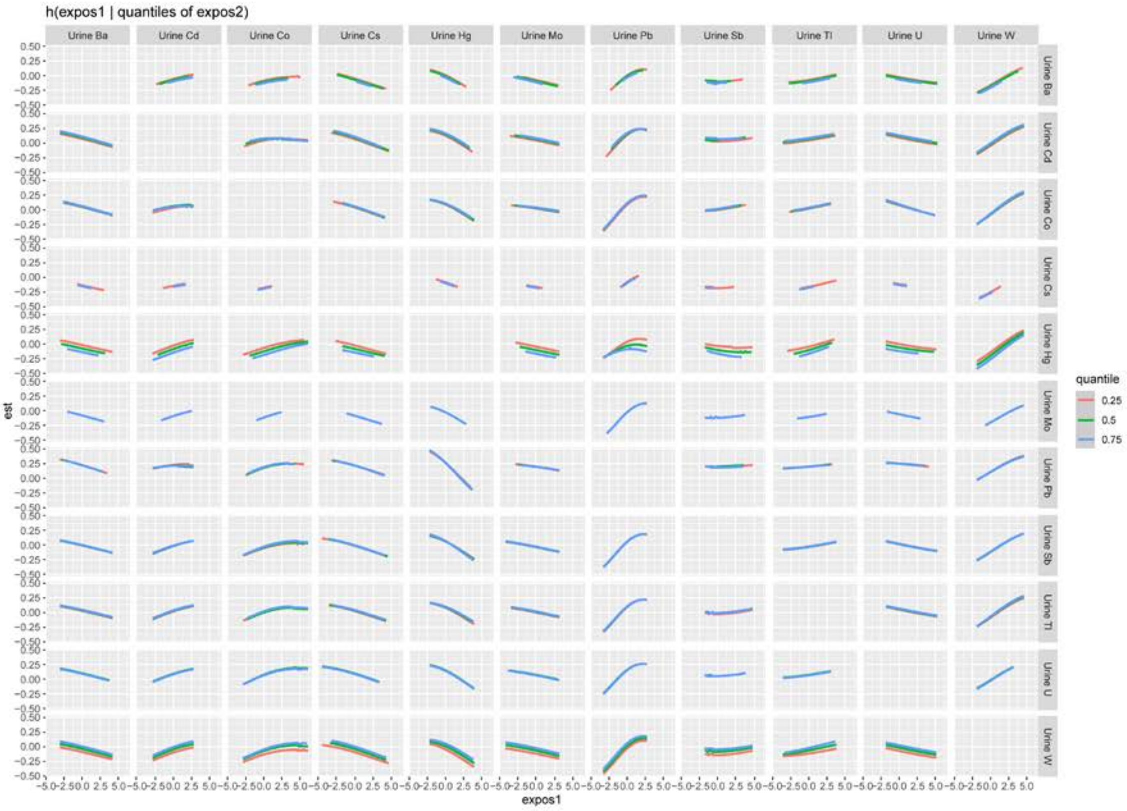

Figure S4. The interaction of urine multiple metals on total IgE. The total IgE was continuous variables (A) and categorical variables (B).
